# Evaluation of Transcutaneous Electrical Nerve Stimulation as a Treatment of Neck Pain due to Musculoskeletal Disorders

**DOI:** 10.4021/jocmr2010.06.370e

**Published:** 2010-06-15

**Authors:** Mikhled Maayah, Mohammed Al-Jarrah

**Affiliations:** a Department of Physiotherapy, Applied Medical Sciences, Jordan University of Science and Technology, Irbid, Jordan

## Abstract

**Background:**

This study was designed to evaluate transcutaneous electrical nerve stimulation (TENS) as a treatment for neck pain due to musculoskeletal disorders within the context of a physiotherapy treatment.

**Methods:**

Thirty subjects with neck pain were randomly allocated to two groups, treated with either TENS (n = 15) or placebo (n = 15). Each subject received one session for one hour. All subjects were evaluated before, during treatment, after switch off and again a week after by using Myometer machine. All subjects completed the follow-up assessment. Subjects referred for out-subjects' physiotherapy department, fulfilling the inclusion and exclusion criteria, took part in the study.

**Results:**

The assessments were compared and used to measure outcome treatment. Improvement in their condition was measured in terms of a reduction in the individual's level of pain during the week after the end of the first session. At the end of the first session, the study showed that 11 subjects (73%) in the treatment and 7 subjects (43%) in the control groups had gained marked improvement. These results are statistically highly significant, (P = 0.01) at the end of the follow-up assessment.

**Conclusions:**

A conclusion could be drawn that a single intense TENS treatment is an effective treatment for neck pain due to musculoskeletal disorders. On the other hand, TENS showed an effective pain relief with subjects who have a mild neck pain rather than those with severe symptoms.

**Keywords:**

Musculoskeletal disorders; Transcutaneous electrical nerve stimulation; Neck pain

## Introduction

Neck pain is the second largest cause of time off work, after low back pain [1]. Pain is a major complaint of subject with neck pain due to musculoskeletal disorders of the cervical spine [2]. Neck pain from musculoskeletal disorders again tends to be worse in the morning and evening, with improvement during the day. This pain often radiates to the shoulder, between the shoulder blades and up the neck to cause headaches [3]. The most commonly prescribed intervention for the treatment of neck pain by general practitioners is rest, followed by analgesics [4, 5]. Neck pain is one of the most common conditions for referral to a physical therapist. Despite the prevalence of neck pain, there is a lack of evidence for commonly used rehabilitation interventions [6].

There is various treatment options used to treat neck pain [7], for example, heat, massage, manipulation, cervical traction and supply of a cervical collar due to musculoskeletal disorders. Among them, transcutaneous electrical nerve stimulation (TENS) is widely available in Western chronic pain clinic [7]. Subjects may experience some relief from pain from these modalities but this improvement is rarely sustained, since subjects frequently return to the physicians without the problem solved. There would therefore appear to be a need for a means of controlling chronic neck pain.

TENS is a simple, noninvasive modality in physiotherapy that is commonly used to control both acute and chronic pain arising from several conditions [8-12]. It was introduced into clinical practice in 1972 as an adjunct to other pain therapies. The mechanism of the action of TENS is still not completely understood. Analgesia may be produced by the modulation of nociceptive input in dorsal horn of the spinal cord by peripheral electrical stimulation of large sensory afferent nerves. This is the 'gate control theory' of pain [13]. Alternatively, electrical stimulation of certain receptor sites in the dorsal horn of the spinal cord may release endorphin, in turn, producing analgesia that can be reversed by naloxone [14, 15].

Several studies examined the efficacy of TENS in musculoskeletal disorders have been published. Since the 1970s, TENS has been gained popularity used as a treatment of acute and chronic pain [11, 16-18]. Transcutaneous electrical nerve stimulation currently is one of the most commonly used forms of electroanalgesia [7]. In medicine, TENS is the most frequently used electrotherapy for producing pain relief.

A number of clinical studies exist concerning the use of TENS for various types of disorders such as low back pain [19-24], Myofascial [25] and arthritic pain [26], sympathetically mediated pain [26-28], bladder incontinence, neurogenic pain [29-32], visceral pain, and postsurgical pain [15, 33, 34], chronic musculoskeletal pain [18, 35].

The chief advantage is that it is a non-invasive and non-toxic form of pain management, which is based, in part, on the Gate Control Theory of pain [36]. It is thought to activate the large diameter, myelinated A-beta fibers which have a low threshold for electrical stimulation [37].

## Patients and Methods

### Subjects

Outpatients with neck pain were recruited from physiotherapy lab in Applied Medical Sciences at Jordan University of Science and Technology. The subjects had been clinically and radiologically diagnosed of neck pain due to musculoskeletal disorders. Further inclusion criteria for the study were aged between 20 to 75 years, neck pain existed most days in the last month. The subject should have received no treatment for neck pain other than oral analgesia for the duration of one week after the end of the first session. And also subject should have had no previous TENS treatment.

Patients were excluded if they had any of the following: with a cardiac Pacemaker, since electrical impulses of the TENS may inhibit action; with history of malignancy, which could be a current cause of bone pain.

On arrival in the department, the study was explained to eligible subjects with neck pain and written informed consent and permission to enter individual subjects was obtained. According to a block randomized allocation table (generated by sample size 2.0 Int), the enrolled subjects were allocated to either the TENS group or the placebo group.

### Design

The design of this study was randomly controlled clinical trial using a block-randomized procedure. Each subject received one session for one hour. All enrolled subjects were evaluated before, during treatment, after switch off and again a weak after by using Myometer machine.

### Treatment

#### Control group

The control group involved a TENS stimulator in working order, using a different set of leads in which contact was broken at the wire connecting the jack plug and the electrode pads. Thus, no stimulation passed to the subject's skin, although a small green light flash continued at the set pulse-rate, reinforcing treatment. This mock TENS had no physiological effect.

#### TENS group

The TENS group subjects received one-hour treatment at the maximum tender area was marked with indelible ink to insure that all measurements were taken at the same point. The pulse-rate or frequency and amplitude or voltage is adjustable and a green light flashes at the selected pulse-rate. Two conductive silicone polymer electrodes were used for stimulation, attached to the TENS machine by a two cord lead, and to the skin with karaya pads electrodes. The karaya pad are conductive and once moistened become tacky allowing the fixation of the electrodes without either jelly or tape. The subject was told that the electrodes were placed primarily over tenders but where pain was diffused, the local acupuncture points around the neck were chosen for placement. Procedure was noted for each subject, but placement varied when painful areas altered. When the electrodes were in place, the investigator turned on the machine. The intensity setting was regulated by the subjects own comfort level and subjects were instructed to indicate when the level of stimulation was at a comfortable and tolerable level. The subjects were instructed that the sensation should not be painful. Adjustments in the intensity were made during the session to maintain it at the same tolerable level. The frequency of the output was set at 4 - 8 Hz and current intensity was raised until the subject reported that it was unpleasant.

The subject was told that the TENS might or might not be effective and that the neck pain medication would be available as needed. All subjects reported a tolerable level of stimulation between 3 and 5 on the amplitude dial and the frequency 2 Hz. The investigator was available thought the sessions to discuss any functional difficulties related to neck pain due to musculoskeletal disorders.

### Evaluation

Primary outcome measures were: (1) Self-assessment forms completed by the subject and the investigator; (2) Daily drug intake and daily pain level were recorded on a diary by the subject; (3) Self-assessment form to evaluate overall weekly pain, sleep disturbance and immediate response to treatment were recorded by the subject; (4) Neck pain measured by Myometer machine. The Myometer scores were measured immediately before the first treatment and subsequently during treatment, after switch off and again a week after the completion of the first treatment. The subject removed sufficient clothing to expose the cervical area and shoulder and sat on a chair. The Myometer was placed centrally, as far as possible, over the maximum tender area. The pressure applied by the investigator was indicated on a pressure transducer in kg - force and was gradually increased until a distinct painful sensation was elicited. This was taken as the pain threshold for that tender area.

## Results

### Subjects

Thirty subjects were recruited, 15 females, 15 males, aged 21 - 70 years with mean of 55.7 years. Concerning duration of the neck pain problem, 48% of the subjects reported having experienced mild gain for more than 5 years, with 3% of the subjects had experienced severe pain for 7 months, and 20% indicating their pain had been quite severe for 5 years and 29% with a vague history of pain.

### Group comparability

After separate randomization for male and female subjects, both groups contained equal numbers of subjects, (n = 15). The treatment group contained 8 female and 7 male subjects, and all completed the trial. The control group contained 7 female and 8 male subjects and all completed the trial.

### Age of subjects in relation to treatment

The range of ages in the two groups was not similar: 23 - 70 years in the treatment group and 35 - 72 years in the control group. The mean age of the treatment group was 53.53 years compared to 58.2 years in the control group. The distributions of age in the two groups are shown in Table 1.

**Table 1:** Characteristics of the Demographic Data of the Study

		Control Group	Treatment Group
Sex	Male	8	7
	Female	7	8
Age of subjects in relation to treatment	Range	35 - 72	23 - 70
	Mean age (SD)	58 (8)	53 (7)
	Mean Female Age (SD)	60 (9)	48 (6)
	Mean Female Age (SD)	56.50 (7)	59 (8)
Symptoms	Left	8 (53.30%)	6 (40%)
	Right	4 (26.70%)	6 (40%)
	Both	3 (20%)	63 (20%)
Severity of Symptoms	Range for the all	11-35	11-35
	Mean score for the all	19.66	19.66
	Mean (SD)	21.47 (1.43)	17.20 (1.15)
Analgesic Intake	No analgesic drugs	8	10
	Simple analgesia only	7	5

### Symptoms

All subjects had a single diagnosis of neck musculoskeletal disorders. The symptoms were for 8 subjects on the left side in the control group and for 4 subjects on the right side, the ratio of left to right was 2:1. The symptoms of pain in the treatment group are equal in left and right, and in both sides in both groups were equal (see Table 1). Although the left side of the neck, is the most common involvement area was in the control group. The duration of symptoms of musculoskeletal disorders was found to be greater among the treatment group.

### Severity of symptoms

Subjects were not divided into groups relating to symptom severity at the beginning of the trial. Although the method employed here is not a standard statistical procedure, the results obtained would appear to agree with the investigator's subjective clinical opinion about each subject. The distribution of the total average of pain scores the week after the end of the first session in the two groups are shown in Table 1.

### Analgesic intake

A record of analgesic intake was taken by each subject for one week after the end of the first session. This was to allow subjects to become stabilized on any drug they had recently been prescribed and also to establish the weekly score after the end of the first session. The type of drug for each subject was recorded. They were discouraged from altering the type of drugs they were taking, but they were encouraged to reduce the amount of analgesia where this was considered to be appropriate.

Subjects were either taking simple analgesia, such as Paracetamol or Brufen. No stronger analgesia was used by any subject during the week except one subject who was taking a strong analgesic for other medical purposes. A record of these was also kept. Table 1 indicates the intake of analgesia in the two groups.

### Comparability of treatment and control groups

For the purposes of this study, it was necessary to obtain two similar groups of subjects so that the effects of treatment could be clearly evaluated and other variables excluded as far as possible.

The TENS and MTENS treatment groups appeared to be broadly comparable in sex, age, duration of present attack, and severity of pain they had experienced before treatment. Additionally, no subject was receiving any physiotherapy treatment for neck musculoskeletal disorders except oral medication. The treatment and control groups therefore seemed to be similar for the variables analyzed. All the subjects received one session of 60 minutes duration. All subjects received similar instructions and explanations prior to beginning the trial. All subjects completed self-assessment forms, and all measurements were carried out by the investigator. It was therefore assumed that any variation in the results was obtained from the effects of TENS.

### Summary of data collection

Information concerning the efficacy of TENS treatment was obtained from subjects as the following:

1. Self-assessment forms completed by the subject and the investigator.
2. Daily drug intake and daily pain level were recorded on a diary.
3. Self-assessment form to evaluate overall weekly pain, sleep disturbance and immediate response to treatment.
4. Neck pain measured by Myometer machine.

### Results of data collection

**Figure 1.**
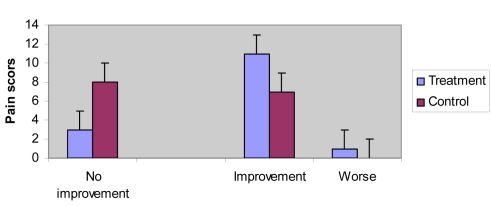
Results of treatment at one week after the first session.

Initially results were analyzed to determine success in relief of pain. All information used in the analysis of treatment was obtained from the self-assessment forms. The week after the first session, 11 subjects in the TENS group and 3 subjects in the control group showed a marked improvement. One subject in the treatment group was considered to be worse after the first three days after the end of the first session (Table 2 and Fig. 1).

**Table 2:** Result at One Week After the First Session

	No improvement	Improvement	Worse
Treatment	3 (20%)	11 (73.33%)	1 (6.67%)
Control	8 (53.33%)	7 (46.67%)	0 (-)

### Short-term of pain relief

Following the end of the first session, no further treatment was given to any subject. After one week, all subjects were followed up to reassess their condition. During the follow-up period, subjects continued to keep a daily record of their drug intake and pain levels. The subjects completed a final self-assessment form on the follow-up visit

Of the all subjects in the treatment group who showed marked pain relief after one week from the end of the first session, 2 showed marked pain relief during the treatment and 2 showed marked improvement after 30 minutes to 2 hours. Of the 8 subjects in the control group who showed no marked pain relief during the session, 4 showed marked pain relief after more than 2 hours and the other 3 subjects showed marked pain relief after 30 minutes. Table 3 shows the duration of short-term pain relief following cessation Of TENS.

**Table 3:** Duration of Short-term Pain Relief Following Cessation of TENS

	Control group	Treatment group
Number of subjects with no marked pain relief	8 (53.33%)	2 (13.33%)
Number of subjects with marked pain relief after 0.5 - 2 hours	3 (20%)	2 (13.33%)
Number of subjects with marked pain relief after more 2 hours	4 (26.67%)	11 (73.33%)

### Statistical analysis of results

**Figure 2.**
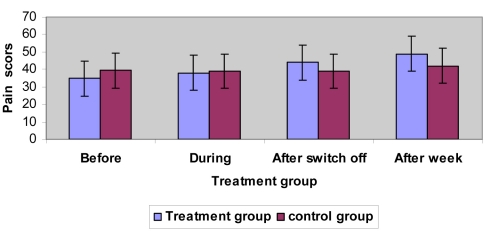
Results of pain threshold measurements for both groups.

No statistical test was carried out for the results after the end of the first session. The difference in response to treatment between the treatment and control groups were obviously statistically significant. After one week of treatment, 11 (73%) subjects in the treatment group had showed marked pain relief compared to 7 (47%) subjects in the control group. The following statistics showed pain threshold measurements for both groups (Table 4). The mean pain threshold before the session in the treatment group is less than the control group (Table 4 and Fig. 2).

**Table 4:** Pain Threshold Measurements for Both Groups

	RANGE	Mean (SD)
Treatment group		
Before	15 - 57	34.80 (1.78)
During	22 - 61	38.13 (10.35)
After switch off	30 - 65	44.13 (10.04)
After week	29 - 60	49.00 (9.62)
Control group		
Before	22 - 78	39.40 (13.67)
During	22 - 51	39.00 (9.47)
After switch off	25 - 60	40.53 (9.64)
After week	25 - 53	42.07 (9.73)

Mean pain threshold values for each group before, during, after, and after a week are shown in Table 4 and Figure 2. The analysis of variance (ANCOVA) showed a statistically significant interaction between groups and before, during, after switch off and after a week follow-up (Table 5), therefore, a statistically significant difference existed among the groups in the terms of mean before and week after pain threshold changes. Using ANCOVA showed statistically significant difference (p < 0.01) between the time (before and 1 week after) and treatment for both groups (Table 5).

**Table 5:** Analysis of Variance for Pain Threshold

Source	F	MS	SS	DF
Time	12.16	420.83	1262.5	3
Subject	9.77	337.86	9798	29
Treatment		60.21	60.2	1
Resid. Between Patient		347.58	9732.2	28
Time*Treatment	5.365	185.59	556.8	3
Error		34.59	2905.5	84
Total			14522.8	119

## Discussion

The results of this study were statistically significant, and especially show that TENS diminishes the pain experienced in the cervical area due to musculoskeletal disorders. The comparison of the effects of TENS and the effect of the placebo was dependent upon the similarity of the subjects in the two groups.

The present study demonstrated that TENS treatment was effective in pain relief. TENS is a popular modality for treating musculoskeletal pain [38]. TENS excited large-diameter afferent fibers [13]. According to the gate control theory [39], TENS may stimulate the large-diameter afferent fibers, which may reduce the transmission of pain signals through the small nociceptive afferent fibers, thereby inhibiting pain discrimination and perception.

### Symptom severity

Little is known about the effects of TENS on similar condition of varying severity. It has been suggested that the greatest pain relief is reported after TENS in subjects who have received minimal previous medical treatment [7].

However, it is possible that those subjects with a long standing history at musculoskeletal disorders could have received a greater number of medical treatments than those with a shorter history of disease, and that they might respond less well to treatment. It was not possible to obtain an accurate history of previous treatment in all cases. It became obvious as the trial proceeded that those subjects with more severe symptoms gained less pain relief than those with mild disease. Following completion of the trial, all the available information regarding factors which might reasonably be associated with disease severity were considered, and subjects were divided into groups (Table 1).

### Use of single-blind methods

Ideally, the investigator should have been blind as to which treatment each subject was receiving. The use of mock electrical 'stimulation' meant that stimulation levels were preset and no adjustment was necessary. It was therefore impossible for the investigator to be "blind" to treatment. Every attempt was made to ensure that treatment procedures were the same for each subject.

### Self-assessment

The difficulty in accurately assessing pain [40] is well known, especially using a practical method in a busy clinical situation, yet such a system is essential for the evaluation of methods controlling pain.

The word pain refers to an endless variety of quantities that are categorized under one label, and not to a single specific entity [40, 41]. A number of methods have been designed to evaluate uncontrolled chronic pain.

It is described by the author as being sufficiently sensitive to detect differences among different methods of treating pain, and has been successfully employed to measure changes in pain quality and intensity produced by TENS [42]. However, the questionnaire is long, detailed and is not a practical tool within the confines of a busy department. It has already been suggested that a subjective measurement of the greatest importance, since only the subject experiences the painful sensations.

### Analgesia

During the week after the end of the first session subjects were asked not to alter their drug which they were taking, but to reduce their analgesic intake whenever it was possible and appropriate. Only one subject altered of type of the drug during the subsequent week. Ideally all subjects should have been prescribed the same type of analgesic, but this was not possible within the time available and within the methodology of the trial.

### Application of the trial results

The result of this study suggests that TENS is an effective means of relieving pain in the neck due to musculoskeletal disorders. TENS was used in the context of a physiotherapy treatment. The results show a statistically significant result in the treatment group while no improvement was shown in the control group. It would seem that TENS could usefully be employed in the treatment of some cases of musculoskeletal pain. The use of a non-invasive technique, such as TENS to control musculoskeletal pain could provide a useful alternative to the use of large amounts of potentially harmful analgesic drugs, especially if sustained pain relief can be achieved.

The result of this study stated that in some conditions it is possible to gain much marked pain relief for up to one week following the end of the first session. There is no certainty whether or not the analgesic effect was sustained after this time, since no further follow-up was attempted. However the method of the application of TENS in this study was flexible. Treatment was limited to one session. Prolonged courses or more frequent application of treatment may produce marked pain relief in greater percentage of subjects.

The immediate short-term or long-term analgesic effect of TENS could also be usefully employed in treating musculoskeletal disorders, in conjunction with other physiotherapy modalities such as traction and mobilization. Most TENS machines are battery operated and portable, which the subjects are able to move whilst the electrodes are in place. Minimal side-effects and the simplicity of the TENS machines is appropriate as a home or self-treatment modality.

The TENS units are also relatively inexpensive. For the above reasons, any subject could buy one and with some instruction by a physiotherapist, could use it safely. Subjects could therefore purchase a machine for use at home for long-term pain relief. This could have positive effect upon Health Services Finance as this alternative might reduce the need for out subject attendances for physiotherapy and consumption of expensive drugs. The use of TENS as a treatment for musculoskeletal disorders of the neck symptoms could completely relieve severe pain in some cases. TENS could also be effective in treating other joints affected by musculoskeletal disorders or in other forms of arthritis.

### Group comparability

The analysis of characteristics of the two groups, showed them to be similar. Both treatment and control groups contained 15 subjects, male and female. The mean age of the control group was greater than that of the treatment group, but age is not thought to affect the response to treatment. Age distribution of subjects in the two groups was similar (Table 1), and analgesic intake was comparable in the two groups, (Table 1). Duration of the symptoms was slightly greater in the control group, although a small proportion of the treatment and control group had bilateral symptoms (Table 1). The two groups were thought to be comparable for symptom severity (Table 1). The similarity of the two groups meant that any variation in their response to treatment could reasonably be attributed to the treatment they received. Therefore differences shown in the result between the treatment and control groups can be assumed to be associated with the effects of TENS treatment.

### Result of treatment

Self-assessment forms and daily analgesic intake diaries and degree of sleep disturbances for each subject were used to obtain a pain score one week after the end of the first session. One week after the end of the first session, 12 (80%) subjects in the treatment group, 7 (47%) subjects of the control group had shown marked pain relief within the criteria laid down for improvement. This suggests that TENS is effective as a means of relieving musculoskeletal disorders pain in most cases, substantially supporting the initial hypothesis.

The result also describes the short-term effects of TENS and suggests that TENS can produce sustained relief from musculoskeletal disorders pain. However, 73% of subjects in the treatment group reported an immediate response to TENS. Although this 'long-term' relief of pain is not analyzed or described in any detail in this study, it is an important feature of TENS. Nevertheless the short-term relief of pain has been demonstrated in both treatment and control groups as 67% of subjects reported an immediate response to treatment. The psychological effect of receiving treatment or attending the physiotherapy department may have contributed to this, as may contact with the therapist. All subjects were aware that they were taking part in a research project and this might have influenced the result.

The results obtained from this study are statistically significant at the 5% level, as they suggest that TENS is more effective than placebo in relieving musculoskeletal disorders pain experienced in the neck. The small number of subjects in the study sample can only be supposed to be representative of the population of subjects with musculoskeletal disorders.

### Relevance of trial methodology

The over all objective of the trial was to evaluate the efficacy of TENS as a means of relieving musculoskeletal pain in the neck. In order to achieve these objectives, a single blind, randomized controlled clinical trial was selected.

### TENS versus placebo

The comparison between TENS and mock electrical stimulation has been evaluated as a valid method of determining the effects of TENS [41]. In order to distinguish the real effect of TENS from that of the placebo effect incurred in a physical treatment, a machine giving mock TENS was used as a control treatment. Also, by using a machine of similar appearance, with identical treatment procedure, any difference in response between two comparable groups can reasonably be attributed to the electrical stimulation, and not to the placement of electrodes on the skin or the use of a previously unknown treatment or treatment procedure.

Finally, the treatment and placebo machines were almost one machine has used, with one producing a 'tingling' sensation effect and the other producing no abnormal sensation on the skin. It was therefore decided that the most appropriate method of evaluating the effect of TENS as treatment for musculoskeletal disorders, was to compare its effects with those of a placebo in two comparable groups of subjects. It was difficult to obtain similar groups of subjects within the time available to conduct the trial. Subjects were considered for entry as they were referred to the department, and it was not possible to match subjects for symptoms and age or to compare the two departments.

### Conclusions

The present study demonstrated that TENS has shown an effective means of providing a sustained pain relief in terms of Myometer machine in subjects complaining from neck pain due to musculoskeletal disorders. On the other hand, TENS may show an effective pain relief with subjects who have a mild pain. TENS is a useful physiotherapy technique in the management of musculoskeletal disorders, but also has a potential use in primary medical care as a means of home treatment where pain relief can not be sustained by drugs. TENS could be used as pain relief to reduce the amount of analgesic drugs taken by subjects, especially for prolonged symptoms.

In comparison with other physiotherapy equipment, the TENS stimulator is compact, cheap, simple to administer, relatively safe to use and free side effects. The pain relief obtained by using TENS does not always last as long as subject would like. It can vary from a few weeks to a few months up to several years.
